# The Influence of Time on the Epidemiology and Clinical Manifestations of Behçet’s Disease in Brazil

**DOI:** 10.3390/jcm12227008

**Published:** 2023-11-09

**Authors:** Lilian T. Hirata, Carlos Eduardo G. Teixeira, Eduardo P. Magalhaes, Ana Paula T. Del Rio, Ibsen Bellini Coimbra, Zoraida Sachetto

**Affiliations:** Unit of Rheumatology, Department of Orthopedics, Rheumatology and Traumatology, School of Medical Sciences, State University of Campinas (UNICAMP), Campinas 13083-855, Brazil; lihtiemi@hotmail.com (L.T.H.); carlosgarcezt@gmail.com (C.E.G.T.); dreduardomagalhaes@gmail.com (E.P.M.); aptdelrio@hotmail.com (A.P.T.D.R.); zoraida@unicamp.br (Z.S.)

**Keywords:** Behçet’s disease, vasculitis, epidemiology, clinical manifestations

## Abstract

Objective: Modifications in the severity and clinical expression of Behçet’s disease (BD) have been described in some areas that are considered endemic for the disease. This study aims to evaluate the chronological changes in epidemiology and clinical characteristics of BD patients in a referral center in Brazil, which is considered a non-endemic area for the disease. Methods: A descriptive and cross-sectional study involving BD patients divided into two groups: group 1 patients were diagnosed and followed between 1988 and 2010, and group 2 were diagnosed and followed between 2011 and 2022. Results: No significant differences were found regarding gender and age at onset of symptoms between groups. We found a significant decrease in the frequency of bilateral ocular involvement, posterior uveitis, and retinal vasculitis. Conclusion: The demographic dates of this group of Brazilian BD patients remained similar over the last decade. Our study supports the notion that BD is becoming lighter in some regions. BD is a severe blinding disorder, and we found a lower frequency of ocular involvement over time. These findings may be attributed to a higher level of education of patients and a growing awareness of the disease. Newer immunomodulating and biologic agents may offer an improved prognosis in patients with BD with severe manifestations.

## 1. Introduction

Behçet’s disease (BD), initially reported by Hulusi Behçet in 1937, is a heterogeneous disease characterized by recurrent mucocutaneous ulcers that can affect multiple organ systems [1,2]. The main symptoms are related to inflammation involving the eyes, skin, mucosa, brain, intestine, and vessels of a small, medium, or large size [1].

BD is reported worldwide, but has a well-known distinct geographical distribution, with higher prevalence in countries along the ancient Silk Road. Formal incidence studies are rare for BD [3]. On the other hand, the prevalence of BD shows considerable variability between countries. Iran and Turkey are the countries with the highest prevalence of BD in the world, and the frequency of BD increases from north to south in Europe. BD is relatively rare in South America [4]. In Brazil, there are still no data available on the incidence of BD. A multicentric epidemiological study in Brazil, including patients with vasculitis under follow-up from nine hospital centers, showed that BD is the most frequent primary vasculitis in the country. The study involved seven centers from the southeast and two from the northeast of Brazil and was performed between 2015 and 2017 [5].

BD usually manifests during the second or third decade of life. Although rare, it can also affect children and older patients [4], and it appears that early disease onset is a factor of poor prognosis, similar to that seen in male patients [6]. Men and women are both affected by the disease; however, there is a difference in sex predominance depending on geographical regions. A male predominance has been described in the Middle East, while a female predominance is seen in Japan, South Korea, and Brazil [5,6,7,8].

BD is a genetically complex disease with a multifactorial etiology. In addition to the genetic associations of HLA and non-HLA predisposition and epigenetic influence, environmental factors are believed to contribute to the pathogenesis of the disease, and among these, infectious agents and specific alterations to the microbiome are relevant in the pathogenesis of BD [9]. Several genome-wide association studies have confirmed that HLA-B51 is the strongest genetic susceptibility factor. HLA-B51 has been considered a risk factor for BD in various population groups. The distribution of BD is directly related to HLA-B51 expression levels, with the highest prevalence in Europe, the Middle East, and the Far East, where HLA-B51 is found in more than 15% of individuals, and the lowest prevalence in Africa, Oceania, and South America [7]. In Brazil, the presence of HLAB-51 was considered a risk factor for BD in only one study conducted [5].

In recent decades, epidemiological data showed that the features of BD have changed over time. A decrease in the prevalence, severity, and clinical expressions of BD was described, mainly in endemic areas [10,11,12,13]. A recent study carried out in Tunisia showed a decrease in the frequency of posterior uveitis between the two decades from 1995 to 2017 [14]. An older study comparing the response to eye treatment over three decades, from 1962 to 2004, carried out in Maryland, USA, showed a definitive trend toward the improvement of clinical outcomes between the 1960s and 1990s [11]. No studies have evaluated these epidemiological changes, especially in South American countries such as Brazil. This study aimed to assess the evolution of epidemiological features and the frequency of clinical manifestations between BD patients divided into two groups according to the follow-up time.

## 2. Materials and Methods

**Study Type**: This is a descriptive and comparative cross-sectional study.

**Ethical considerations:** This study was conducted in accordance with the Helsinki Declaration and was approved by the local ethics committee of the School of Medical Sciences of the State University of Campinas—UNICAMP (CAAE number: 59537422.3.0000.5404).

All participants provided written informed consent before the study.

**Patients**: We selected medical records of all BD patients with regular visits to the Rheumatology unit outpatient clinic at the Hospital of Clinics of the University of Campinas (HC/FCM-UNICAMP) from 1988 to 2022. The patients were divided into two groups according to the date of their first visit. Group 1 consisted of patients diagnosed and followed between 1988 and 2010 (data already published by Sachetto et al. [15]), and group 2 included patients diagnosed and followed between 2011 and 2022. The data used for group 1 were collected in 2010, and those for group 2 were collected between October and November 2022. All of the manifestations present at the end of the period were analyzed. At the time of data collection, the patients in group 1 had an average of 14 years of disease, and patients in group 2 had an average of 13 years of disease. In both groups, the patients had been followed up for an average of 9 years. All patients fulfilled the 1990 International Study Group (ISG) diagnostic criteria [16]. All patients were classified as having the complete form of BD. BD patients who were 18 years or older and consented to participate were included. Records were analyzed for demographic data, disease history, and clinical manifestations. Disease onset was assumed at the time of the first symptom occurrence. Ocular involvement was considered when uveitis or retinal vasculitis had been diagnosed by an ophthalmologist. Neurological involvement was analyzed in conjunction with a neurologist after the exclusion of different causes. Vascular involvement was considered in the presence of deep or superficial venous thrombosis, arterial thrombosis, arterial aneurysm, and arteritis. Gastrointestinal involvement was demonstrated via endoscopy, colonoscopy, or contrast-enhanced imaging. Pathergy testing was not performed.

This study also presents the descriptive data of patients diagnosed from 2011 to 2022 (group 2).

**Statistical analysis**: Statistical analysis was performed with SAS (Statistical Analysis System) version 9.4 for Windows. Age distribution was expressed as mean ± standard deviation. Results between the groups were compared using the t-Student test (Satterthwaite method). Association tests and proportion comparisons were performed using Pearson’s chi-square test or Fisher’s exact test. The level of significance was 5% or *p* < 0.05.

## 3. Results

### 3.1. Descriptive Analysis of Group 1

Eighty-seven patients were described in group 1. There were 47 women and 40 men (sex ratio F/M, 1.18/1). The mean age at BD onset for women was 28.59 ± 7.86 (range, 8–45 years), and for men, it was 27.37 ± 7.25 (range, 12–46 years). The average delay between the onset of the symptoms and diagnosis of BD was three years. The frequencies of clinical manifestations in group 1 (n = 87) were as follows: oral ulcers in 100% (N = 87), genital ulcers in 77% (N = 67), pseudofolliculitis in 47,67% (N = 41), erythema nodosum in 19,8% (N = 17), vascular involvement in 13.95% (N = 12), and gastrointestinal involvement in 1.16% (N = 1). Central nervous system involvement was present in 31% of the patients (N = 27) as one of the initial manifestations of the disease. Ocular involvement was the first disease manifestation in 23 patients (47.9%). Ocular manifestations were observed in 70 patients (80.5%), panuveitis in 65 (93%), and retinal vasculitis in 40 (57%) of the patients with ocular involvement.

### 3.2. Descriptive Analysis of Group 2

Forty-eight patients were evaluated in group 2. There was a predominance of women (sex ratio F/M 1.4/1). The mean age at disease onset was 29.3 ± 9.38 (range, 13–50) years and 30.1 ± 10.74 (range, 0.58–49) years in women and men, respectively. The frequencies of clinical manifestations in group 2 (n = 48) were as follows: oral ulcers in 97.9% (N = 47), genital ulcers in 81.3% (N = 39), ocular manifestation in 62.5% (N = 30), pseudofolliculitis in 35.4% (N = 17), erythema nodosum in 18.8% (N = 9), vascular involvement in 18.8% (N = 9), and gastrointestinal involvement in 6.3% (N = 3). Central nervous system involvement was present in 14 patients 29.2% (N = 14), and in all of them, this was one of the initial manifestations of the disease. Table 1 describes the demographic and clinical data in groups 1 and 2; ocular involvement was the first disease manifestation in 23 patients (47.9%). Patients with ocular lesions (N = 30) had the first symptom at the age of 30 ± 1.61 years, while those without ocular lesion (N = 18) were 29.09 ± 2.86 years. Female patients had a higher risk of genital ulcers (*p* = 0.002; OR 18) and erythema nodosum (*p* = 0.06; OR 7.6). Also, these manifestations were more common in women. Men were more likely to present CNS (*p* = 0.04; OR 3.8) involvement and retinal vasculitis (*p* = 0.04; OR 4). These sex-specific differences between men and women are described in Table 2.

The presence of genital ulcers was associated with retinal vasculitis (*p* = 0.0321). Ocular lesion occurrence was associated with pseudofolliculitis (*p* = 0.0238), vascular (*p* = 0.0056), and neurological involvement (*p* = 0.0018). Patients with bilateral ocular involvement showed an association with the presence of retinal vasculitis (*p* = 0.0117), posterior uveitis (*p* = 0.0114), and intermediate uveitis (*p* = 0.0331) and CNS involvement (*p* = 0.0455). Ocular involvement as the initial manifestation of the disease was associated with bilateral ocular involvement (*p* < 0.0001), vascular involvement (*p* = 0.0238), CNS involvement (*p* = 0.0184), and thrombophlebitis (*p* = 0.0507). The presence of posterior uveitis was associated with CNS involvement (*p* = 0.0053). Finally, the presence of vascular manifestations was associated with CNS involvement (*p* < 0.0001).

The associations between the disease expressions in group 2 are described in Table 3.

### 3.3. Comparative Analysis between Groups 1 and 2

The comparative analysis between groups 1 and 2 showed no difference in sex (*p* = 0.6295) and age at disease onset (*p* = 0.3251). The mean age at disease onset in both groups was 29.7 ± 1.44 (range, 0.58–50) years. There was also no difference in the time of delay in diagnosis (*p* = 0.49). Regarding clinical manifestations, significant differences were found only between the presence of ocular involvement, posterior uveitis, and retinal vasculitis. There was a decreased frequency of ocular involvement, bilateral ocular involvement, posterior uveitis, and retinal vasculitis. These data are described in Table 4.

## 4. Discussion

Despite changes in the pattern of demographic characteristics and clinical manifestations of BD over time having been described, there are few comparative epidemiological studies on BD. Decreases in prevalence, severity, and clinical expression have been found in areas considered endemic for BD [9,15,16,17]. Investigating these changes in different geographic areas or populations is necessary, since it can provide relevant clinical and research information. No studies have evaluated the presence of these epidemiological changes in areas considered non-endemic for BD in South America, especially in Brazil.

In our study, consistent with previous data, there were no significant differences in the age at onset of symptoms between the groups studied [15,16,17], reflecting that the mean age of BD onset remained unchanged during the two periods.

The female-to-male ratio remained stable in that study, which is similar to the data shown by two previous studies [15,17]. On the other hand, a study in Turkey showed an increase in BD in women, with a gradual decrease in the sex ratio [17].

Regarding systemic involvement in our BD patients, we observed an unchanged frequency of mucocutaneous involvement during the disease course, which is also described in a study in Tunisia comparing the pattern of clinical manifestations over two decades [17]. These findings differ from data previously shown by a study in Turkey, which found a decreasing trend in the frequency of genital ulcers in BD [18].

Our results showed that the proportion of ocular involvement, bilateral ocular involvement, and posterior uveitis and the presence of retinal vasculitis decreased in both groups. A retrospective study involving 246 patients in Tunisia showed an increase in the frequency of ocular involvement, which differs from our findings [14]. The presence of posterior uveitis also decreased in the same study over the years [14]. Another retrospective study from Korea involving BD patients from 1983 to 2012 showed a decrease in ocular involvement [17]. An older study involving Japanese patients with BD showed that the number of ocular attacks and visual acuity did indeed improve during the 1990s [13]. We believe that our findings are due to better knowledge of the disease by other medical specialties, as well as increased and more available drugs with greater efficacy in treating this manifestation. Ocular involvement is considered a severe manifestation of BD. The severity of involvement was not assessed in this study.

The major limitation of this study is that it was carried out with data from a reference hospital. A likely bias is the absence of patients followed up in basic health units and private practices. On the other hand, patients with severe manifestations are generally referred to tertiary centers. A positive point of our study is the significant similarity between the average time of illness and the average follow-up time of the patients, at the time of data collection, between the two groups studied. Given this, we believe that the data presented in this study are representative, as they reflect the reality of a group of patients with severe and rare vasculitis followed in a reference hospital covering about 86 municipalities with approximately 6.5 million inhabitants.

This hospital-based study supports the notion that BD is becoming milder in some regions. BD is a severe blinding disorder, and we found a lower frequency of ocular involvement, as well as of the most serious ocular manifestations. We attribute these findings to the introduction of more potent corticoid-spare agents and targeted agents and possibly to better propaedeutic tools for the earlier identification of ocular involvement.

## 5. Conclusions

The demographic data of this group of Brazilian BD patients remained similar over the last decade. Our study supports the notion that BD is becoming milder in some regions. BD is a severe blinding disorder, and we found a lower frequency of ocular involvement over time. These findings may be attributed to a higher level of education of patients and a growing awareness of the disease. Newer immunomodulating and biologic agents may offer an improved prognosis in patients with BD with severe manifestations.

## Figures and Tables

**Table 1:** Demographic and clinical data in each studied group.

	Group 1		Group 2	
	N = 87		N = 48	
	N	%	N	%
Female	47	54	28	58.3
Male	40	46	20	41.7
Age at disease onset in women, years	28.59		29.3	
Age at disease onset in men, years	27.37		30.1	
Oral aphtosis	87	100	47	97.92
Genital aphtosis	67	77.01	39	81.25
Ocular involvement	70	80.46	30	62.5
Pseudofoliculitis	41	47.67	17	35.42
Nodosum erythema	17	19.77	9	18.75
Polyarthritis	21	24.14	7	14.58
Oligoarthritis	6	6.90	7	14.58
Vascular involvement	12	13.95	9	18.75
CNS involvement	27	31.03	14	29.17
GIT involvement	1	1.16	3	6.25

CNS: central nervous system; GIT: gastrointestinal tract.

**Table 2:** Sex-specific significant differences between men and women—group 2.

	Male N = 20	Female N = 28	OR
Genital ulcer	12	**27**	18
Erythema nodosum	1	**8**	7.6
CNS involvement	**9**	5	3.8
Retinal vasculitis	**8**	4	4

**Table 3:** Clinical symptom associations—group 2.

Genital aphtosis	Retinal vasculitis	*p* = 0.0321
Ocular involvement	Pseudofoliculitis	*p* = 0.0238
	Vascular involvement	*p* = 0.0056
	CNS involvement	*p* = 0.0018
Bilateral ocular involvement	Retinal vasculitis	*p* = 0.0117
	Posterior uveitis	*p* = 0.0114
	Inermediate uveitis	*p* = 0.0331
	CNS involvement	*p* = 0.0445
	Ocular involvement at the disease onset	*p* = 0.0001
Ocular involvement at the disease onset	Vascular involvement	*p* = 0.0238
	CNS involvement	*p* = 0.0184
CNS involvement	Vascular involvement	*p* > 0.0001
	Posterior uveitis	*p* = 0.0053

CNS: central nervous system.

**Table 4:** Comparison of ocular manifestation between groups 1 and 2.

	Group 1 N = 87		Group 2 N = 48		*p* Value
	N	%	N	%	
Ocular involvement	70	80.46	30	62.50	0.0226
Bilateral ocular involvement	55	63.95	21	43.75	0.0236
Posterior uveitis	65	74.71	18	37.50	<0.0001
Retinal vasculitis	40	46.51	12	25	0.0143

## Data Availability

Not applicable.
